# CAPt’n of Actin Dynamics: Recent Advances in the Molecular, Developmental and Physiological Functions of Cyclase-Associated Protein (CAP)

**DOI:** 10.3389/fcell.2020.586631

**Published:** 2020-09-24

**Authors:** Marco B. Rust, Sharof Khudayberdiev, Silvia Pelucchi, Elena Marcello

**Affiliations:** ^1^Molecular Neurobiology Group, Institute of Physiological Chemistry, University of Marburg, Marburg, Germany; ^2^DFG Research Training Group, Membrane Plasticity in Tissue Development and Remodeling, GRK 2213, University of Marburg, Marburg, Germany; ^3^Center for Mind, Brain and Behavior, University of Marburg and Justus-Liebig-University Giessen, Giessen, Germany; ^4^Department of Pharmacological and Biomolecular Sciences, University of Milan, Milan, Italy

**Keywords:** cyclase-associated protein, CAP, SRV2, Cofilin, F-actin, G-actin

## Abstract

Cyclase-associated protein (CAP) has been discovered three decades ago in budding yeast as a protein that associates with the cyclic adenosine monophosphate (cAMP)-producing adenylyl cyclase and that suppresses a hyperactive RAS2 variant. Since that time, CAP has been identified in all eukaryotic species examined and it became evident that the activity in RAS-cAMP signaling is restricted to a limited number of species. Instead, its actin binding activity is conserved among eukaryotes and actin cytoskeleton regulation emerged as its primary function. However, for many years, the molecular functions as well as the developmental and physiological relevance of CAP remained unknown. In the present article, we will compile important recent progress on its molecular functions that identified CAP as a novel key regulator of actin dynamics, i.e., the spatiotemporally controlled assembly and disassembly of actin filaments (F-actin). These studies unraveled a cooperation with ADF/Cofilin and Twinfilin in F-actin disassembly, a nucleotide exchange activity on globular actin monomers (G-actin) that is required for F-actin assembly and an inhibitory function towards the F-actin assembly factor INF2. Moreover, by focusing on selected model organisms, we will review current literature on its developmental and physiological functions, and we will present studies implicating CAP in human pathologies. Together, this review article summarizes and discusses recent achievements in understanding the molecular, developmental and physiological functions of CAP, which led this protein emerge as a novel CAPt’n of actin dynamics.

## Introduction

Cyclase-associated protein (CAP) has been discovered three decades ago in the budding yeast *Saccharomyces* (*S*.) *cerevisiae* as a component of a complex involved in activation of RAS-family GTPase ras-like protein 2 (RAS2), which controls activity of the adenylyl cyclase (AC) and, hence, cyclic adenosine monophosphate (cAMP) signaling (Fedor-Chaiken et al., 1990; Field et al., 1990). Yeast expressing mutant CAP variants suppress a phenotype (heat shock and nitrogen starvation sensitivity) elicited by the hyperactive RAS2 variant RAS2-V19, thus explaining its alternate name suppressor of RAS2-V19 (SRV2; Fedor-Chaiken et al., 1990). However, CAP mutant yeast displayed cellular defects including impaired growth and altered morphology that could not be attributed to defective RAS2-cAMP signaling (Field et al., 1990; Zelicof et al., 1993; Hubberstey and Mottillo, 2002), thereby suggesting an implication in additional cellular processes. Indeed, subsequent studies identified CAP as a multifunctional protein that apart from regulating RAS2 activity is capable of actin binding (Lila and Drubin, 1997; Hubberstey and Mottillo, 2002; Ono, 2013; Zhou et al., 2014a). Since these initial studies, CAP has been detected in all eukaryotic species examined (Ono, 2013). Lower eukaryotic organisms and most invertebrates possess only one CAP, instead some invertebrates [e.g., *Caenorhabditis* (*C*.) *elegans*] as well as vertebrates have two CAP isoforms with tissue specific expression (Ono, 2013). Studies on model organisms led to the assumption that CAP’s activity in RAS-cAMP signaling is restricted to a limited number of species including some fungi and protists (Bahn and Sundstrom, 2001; Hubberstey and Mottillo, 2002; Ono, 2013). However, a recent study suggested an interaction of CAP-actin complexes with AC in human pancreatic cancer cells (Quinn et al., 2017). Instead, CAP’s actin binding activity is highly conserved and present in higher eukaryotic cells (Ono, 2013; Zhou et al., 2014a). Early studies suggested a rather passive role for CAP in actin cytoskeleton regulation, which was believed to act via sequestering globular actin monomers (G-actin; Hubberstey and Mottillo, 2002). This view has changed drastically in the last decade, because CAP has been implicated in almost all steps relevant for actin dynamics, the spatiotemporally controlled assembly and disassembly of actin filaments (F-actin). Specifically, these studies unraveled (i) a cooperation of CAP with key actin regulators such as ADF/Cofilin and Twinfilin in F-actin disassembly including dissociation of actin subunits from filaments’ barbed and pointed ends as well as F-actin severing, (ii) a nucleotide exchange activity on G-actin that is required for F-actin assembly, and (iii) an inhibitory function towards the F-actin assembly factor inverted formin 2 (INF2), and they linked each individual actin activity to specific protein domains (Chaudhry et al., 2013; Jansen et al., 2014; Johnston et al., 2015; Kotila et al., 2018, 2019; Mu et al., 2019, 2020; Shekhar et al., 2019). In this article, we will summarize and discuss important recent progress in CAP’s structure and molecular functions, focusing on those studies that have been published since Shoichiro Ono’s excellent review in 2013 (Ono, 2013). Moreover, we will review current literature on CAP’s manifold developmental and physiological functions, focusing on selected model organisms including *Drosophila* (*D*.) *melanogaster*, *C. elegans* and mouse, and we will present studies implicating CAP in the mechanisms of human diseases. Together, in this review article we will provide a comprehensive overview of CAP’s molecular, developmental and physiological functions.

## Recent Achievements in Structure and Molecular Functions

### Structure and Domain Organization

**FIGURE 1:**
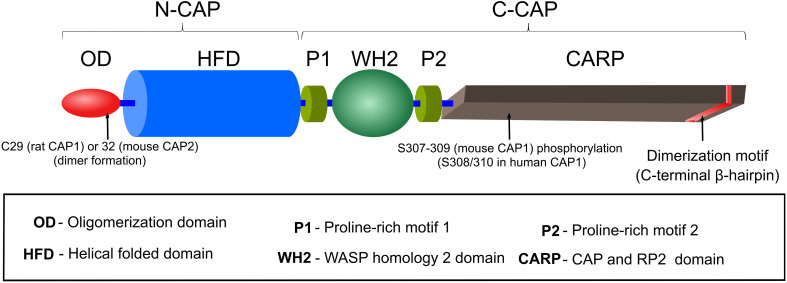
Domain organization of CAP. A detailed description of CAP’s motifs and domains is provided in the section ‘Structure and domain organization’.

Cyclase-associated proteins are multifunctional proteins, composed of 526 amino acids (AA) in yeast and 474 (CAP1) or 476 (CAP2) AA in mouse, and they consist of several distinct motifs and domains, including an oligomerization domain (OD), a helical folded domain (HFD), two proline-rich motifs (P1, P2) separated by a Wiscott-Aldrich-Syndrome protein (WASP) homology 2 (WH2) domain and followed by a domain termed CAP and RP2 (CARP) domain that harbors a dimerization motif at the most C-terminal part of the protein (Figure 1). Crystallization of full length CAP was not possible due to its tendency to form high molecular weight aggregates, possession of autoproteolytic activity and possibly due to ‘unstable’ WH2/P2 domains (Hofmann et al., 2002; Yusof et al., 2005; Kotila et al., 2018). Consequently, only the crystal structures of HFD and β-sheets within the CARP domain have been determined to date (Ksiazek et al., 2003; Dodatko et al., 2004; Mavoungou et al., 2004; Kotila et al., 2018, 2019). In this section, we will provide an overview of the structure and function for each individual CAP domain and motif. The next section comprises a detailed description of CAP’s activities in actin dynamics regulation. Although most of the initial work has been carried out in yeast, we decided to avoid the yeast nomenclature in this section and will refer to SRV2 as yeast CAP to keep a better flow. Further, in both sections, we did not differentiate between individual CAP isoforms.

#### Oligomerization Domain

Oligomerization domain (OD) is composed of the N-terminal ∼40 AA residues, which were predicted to form a coiled-coil structure due to presence of heptad repeat motifs (αXXαXXX), in which α represents a hydrophobic AA (Nishida et al., 1998). This region is sufficient for binding and activation of AC in yeast (Nishida et al., 1998), a function that is absent from CAP in higher eukaryotes (Hubberstey and Mottillo, 2002; Ono, 2013). In comparison to other CAP domains, OD is least conserved among species (Matviw et al., 1992). However, a requirement of this region for oligomerization is conserved from yeast to mammals, and oligomerization enhances CAP’s actin dynamics regulatory potential (Quintero-Monzon et al., 2009; Chaudhry et al., 2013; Jansen et al., 2014). Since in unbound protein OD is either unstructured or in random coil conformation, it has been hypothesized that this region could acquire coiled-coil structure upon AC binding or self-oligomerization (Ksiazek et al., 2003; Mavoungou et al., 2004). A specific role of this domain in oligomerization will be discussed below. Notably, this domain also includes a conserved cysteine residue, which recently was implicated in covalent dimer formation, Cofilin1 interaction and regulation of actin dynamics (Liu et al., 2018; Pelucchi et al., 2020a).

#### Helical Folded Domain

Helical folded domain encompasses AA residues ∼40–220 in (mouse) CAP, which forms an α-helix bundle composed of six antiparallel helices (15–25 AA residues each) that are connected by irregular loops of 5–12 AA residues (Ksiazek et al., 2003; Mavoungou et al., 2004). Although, earlier structural studies proposed that HFD could form dimers (Ksiazek et al., 2003; Yusof et al., 2005, 2006), later it was suggested that HFD/HFD interaction might be rather unspecific and transient and most likely does not contribute to stable dimer formation (Mavoungou et al., 2004; Yusof et al., 2006).

From a functional perspective, HFD binds to complexes composed of G-actin together with either ADF/Cofilin or Twinfilin (Moriyama and Yahara, 2002; Quintero-Monzon et al., 2009; Johnston et al., 2015), and it promotes F-actin depolymerization by interacting with ADF/Cofilin-bound pointed ends or Twinfilin-bound barbed ends (Johnston et al., 2015; Kotila et al., 2019; Shekhar et al., 2019). In addition, HFD interacts with ADF/Cofilin-bound F-actin to promote severing (Chaudhry et al., 2013), but this mechanism seems not to be the major contributor to CAP-dependent F-actin disassembly (Kotila et al., 2019; Shekhar et al., 2019). HFD’s structure is unique, not shared by any other ABP and differs from other actin-binding domains (ABD) in the mode of G-actin binding (Ksiazek et al., 2003; Kotila et al., 2019). Thus it binds efficiently to ADP-G-actin only in complex with ADF/Cofilin or Twinfilin (Moriyama and Yahara, 2002; Quintero-Monzon et al., 2009; Johnston et al., 2015). However, HFD’s interaction interface on G-actin does not overlap with that of ADF-H (actin depolymerizing factor homology domain), the ABD of ADF/Cofilin and Twinfilin, thereby allowing simultaneous binding of CAP, G-actin and ADF/Cofilin or Twinfilin (Kotila et al., 2019). HFD binds to the pointed end of G-actin, between subdomains (SD) 2 and 4. On the contrary, ADF-H binds to the barbed end between SD1 and SD3. It was proposed that binding of ADF/Cofilin induces a slight twist in the actin molecule (Kotila et al., 2019), thereby enabling HFD binding (Moriyama and Yahara, 2002).

#### Proline-Rich Motifs (P1, P2) and WH2 Domain

The central region comprising AA residues ∼220–320 in (mouse) CAP consists of two proline-rich motifs (P1, P2), which are separated by a WASP homology 2 (WH2) domain. Profilin, an ABP that promotes actin polymerization, binds to P1 of yeast CAP *in vitro* and *in vivo* (Bertling et al., 2007). Instead, Profilin can interact with both P1 and P2 domains of mouse CAP (Makkonen et al., 2013). Compared to P1, P2 contains fewer proline residues. It interacts with Src homology 3 (SH3) domain-containing proteins such as actin-binding protein 1 (ABP1) and is required for CAP localization to cortical actin patches in yeast (Freeman et al., 1996; Lila and Drubin, 1997; Balcer et al., 2003). Interestingly, human CAP also interacts with SH3 domain of the tyrosine kinase abelson murine leukemia viral oncogene homolog 1 (ABL) through its P1 region (Freeman et al., 1996). The functional relevance of CAP’s interaction with Profilin or ABL remained elusive, also because P1 mutations only caused very subtle effects in yeast (Bertling et al., 2007). However, genetic studies in *D. melanogaster* revealed at least functional interaction of CAP with Profilin or ABL, e.g., in determining cell morphology or in growth cone function (Wills et al., 1999, 2002; Benlali et al., 2000).

The function of the WH2 domain, located between P1 and P2 and encompassing AA residues ∼250–280, was deduced from its homology to other WH2 containing proteins including type I nucleation promoting factors (e.g., WASP and WAVE), Ena/VASP or β-Thymosin that participate in actin nucleation, F-actin elongation or G-actin sequestering, respectively (Paunola et al., 2002; Dominguez, 2016). Unlike most other WH2 domains that bind ATP-G-actin with higher affinity than ADP-G-actin (Dominguez, 2016), yeast CAP’s WH2 binds both ATP- and ADP-bound G-actin with similar affinity (*Kd* 1.5 μM) (Chaudhry et al., 2010). In comparison to yeast counterpart, mouse CAP’s WH2 possess slightly higher affinity towards ATP-bound G-actin (*Kd* 0.73 μM) (Makkonen et al., 2013). Whether this underlies species specific or experimental difference needs to be clarified. Yeast and mouse CAP’s WH2, together with the adjacent CARP domain (see below), are necessary for efficient catalysis of the ATP-for-ADP exchange on ADF/Cofilin-bound G-actin (Quintero-Monzon et al., 2009; Chaudhry et al., 2010; Jansen et al., 2014). Specific mutations disrupting G-actin-binding of WH2 caused actin disorganization associated with cell growth and cell morphogenesis defects in yeast (Chaudhry et al., 2010). WH2 domain of human CAP might bind to the ABP INF2, but additional CAP domains or interactions are necessary for this function (Mu et al., 2019, 2020). The functional relevance of this interaction is discussed below.

#### CAP and Retinitis Pigmentosa Protein 2 (CARP) Domain

Cyclase-associated protein’s C-terminus, between AA residues ∼320 and 474 possesses a β-sheet structure composed of six coils of right-handed parallel β-strands forming the core of the β-sheet and an additional C-terminal β-hairpin, which extends away from the core and participates in homodimer formation (Dodatko et al., 2004). β-hairpin interacts with the core β-sheet of a second CAP and forms a stable strand-exchanged dimer (Dodatko et al., 2004; Hliscs et al., 2010; Kotila et al., 2018). Since CAP’s β-sheet displays structural similarity to otherwise functionally unrelated proteins including X-linked retinitis pigmentosa protein 2 (RP2) this domain is referred to as CAP and RP2 (CARP) domain (Dodatko et al., 2004). CARP binds ADP-G-actin with high affinity (*Kd* 0.02–0.05 μM) and in 1:1 stoichiometry (Mattila et al., 2004; Makkonen et al., 2013). This interaction is unique in several ways (Iwase and Ono, 2017; Kotila et al., 2018). First, two CARP domains form a homodimer and bind simultaneously two ADP-G-actin, whereby each G-actin interacts with both CARP domains. Second, CARP binds to the pointed end of ADP-G-actin on SD1, SD2, and SD3 and forms the largest binding interface among known ABD (Kotila et al., 2018). These two observations explain the high affinity of CARP, however, only in dimeric form, towards ADP-G-actin (Iwase and Ono, 2016; Kotila et al., 2018). Thus, mutations disrupting CARP homodimer formation abolish interaction with ADP-G-actin and its recharging to ATP-G-actin (Iwase and Ono, 2016). Interestingly, structural analysis also revealed that CARP binding of G-actin could create a sterical clash with Profilin or ADF-H (Kotila et al., 2018). These findings provide an explanation why CARP competes with Profilin and ADF-H-containing ABP such as ADF/Cofilin and Twinfilin and enhances ADF/Cofilin dissociation from ADP-G-actin, thus priming it for nucleotide exchange (Mattila et al., 2004; Chaudhry et al., 2010; Ono, 2013; Johnston et al., 2015; Kotila et al., 2018). Notably, CARP’s binding interface on ADP-G-actin does not overlap with that of HFD, which is in line with the notion that both domains, presumably in cooperation with WH2, can work sequentially in ADF/Cofilin-mediated F-actin depolymerization and G-actin recharging (Kotila et al., 2019; Shekhar et al., 2019).

### Function of CAP in Actin Dynamics

**FIGURE 2:**
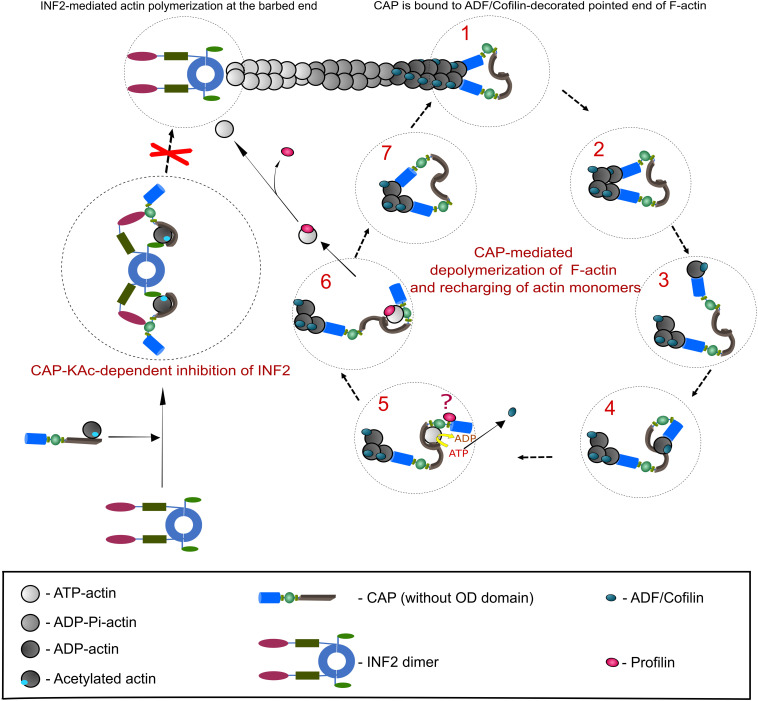
CAP-mediated regulation of actin dynamics. This scheme depicts the best studied CAP-dependent mechanisms relevant for actin dynamics. A detailed description of both mechanisms is provided in the section ‘Function of CAP in actin dynamics’, which also includes additional actin regulatory mechanisms that depend on CAP. Right panel presents a stepwise CAP-dependent depolymerization mechanism of ADF/Cofilin-decorated F-actin pointedends (based on Kotila et al., 2018, 2019). Although CAP forms hexamers, for the purpose of clarity we depicted them as dimers. Additionally, OD was removed. Two HFD (blue) of CAP dimer bind to ultimate and penultimate actin subunit at ADF/Cofilin-decorated pointed end (step 1). HFD thereby destabilizes intermolecular interaction of both actin subunits (step 2). HFD removes ADF/Cofilin-bound ultimate actin subunit from pointedend (step 3) and hands it over to CARPdomain (gray; step 4). Binding of CARP and WH2 (green) domains releases ADF/Cofilin and HFD from G-actin. Next, G-actin is recharged (ADP to ATP) and most likely transferred to Profilin (red) that binds CAP’s P1 motif (step 5 and 6). Question mark in step 5 indicates that the functional relationship between CAP and Profilin is still unclear. Polymerization competent, Profilin-bound ATP-G-actin can now be used for F-actin assembly. Step 7 indicates that a new round of CAP-dependent depolymerisation of ADF/Cofilin-bound actin starts. Left panel: in addition to actin depolymerisation at ADF/Cofilin-decorated pointed ends, CAP can impede F-actin assembly by inhibiting INF2 (depicted as dimer; based on Mu et al., 2019). Binding of a complex composed of lysine-acetylated (KAc-) actin and CAP to INF2’s DID (Diaphanous inhibitory domain, magenta) and DAD (Diaphanous autoregulatory domain, light green) keepINF2 in an inactive state, thereby inhibiting INF2-mediated actin polymerization.

In order to maintain fast F-actin dynamics a tight balance between barbed end polymerization and pointed end depolymerization of F-actin is needed (Pollard, 2016). It has been demonstrated that ADF/Cofilin, in addition to F-actin severing, promotes pointed end depolymerization (Pollard, 2016; Shekhar and Carlier, 2017; Wioland et al., 2017), albeit with a relatively slow speed that cannot explain fast F-actin disassembly in cells (Miyoshi and Watanabe, 2013). Furthermore, upon ADF/Cofilin-mediated depolymerization ADP-G-actin must be removed from ADF/Cofilin and subsequently converted (recharged) to polymerization competent ATP-G-actin, a function which has been dedicated to Profilin (Goldschmidt-Clermont et al., 1991). However, compared to ADF/Cofilin (*Kd* 0.1 μM), the ADP-G-actin affinity of Profilin (*Kd* 1.5 μM) is rather low (Carlier et al., 1997; Vinson et al., 1998), suggesting that additional factors are required to mediate G-actin transition from ADF/Cofilin to Profilin and to convert ADP-G-actin into Profilin-binding favored form, i.e., ATP-G-actin. CAPs are ideal candidates for these functions, since they possess necessary protein domains to (i) efficiently interact with ADF/Cofilin-ADP-G-actin complexes and ADF/Cofilin-decorated filaments’ pointed ends (Moriyama and Yahara, 2002; Quintero-Monzon et al., 2009; Kotila et al., 2019; Shekhar et al., 2019), (ii) displace ADF/Cofilin from ADP-G-actin (Balcer et al., 2003; Mattila et al., 2004; Chaudhry et al., 2014; Kotila et al., 2018), and (iii) exhibit nucleotide exchanging activity on G-actin (Balcer et al., 2003; Chaudhry et al., 2007; Quintero-Monzon et al., 2009; Nomura et al., 2012). Recent studies from Pekka Lappalainen’s lab and from Bruce Goode’s lab (Kotila et al., 2018, 2019; Shekhar et al., 2019), together with previously generated data by the ADF/Cofilin and CAP research community (Ono, 2013), made a significant impact on our understanding how CAP can perform the aforementioned functions *in vitro*, and most likely *in vivo*. Based on this, we will summarize a proposed model for F-actin depolymerization and G-actin recharging carried out by CAP (Figure 2).

#### F-Actin Depolymerizing and G-Actin Recharging

Cyclase-associated protein has two segments, N-CAP and C-CAP, which have specific biochemical activities and can function separately in controlling actin dynamics (Balcer et al., 2003; Mattila et al., 2004; Quintero-Monzon et al., 2009; Chaudhry et al., 2014). N-CAP is composed of OD and HFD (Ksiazek et al., 2003; Mavoungou et al., 2004), which have been implicated in oligomerization as well as F-actin depolymerization and severing (Quintero-Monzon et al., 2009; Chaudhry et al., 2013; Jansen et al., 2014; Kotila et al., 2019; Shekhar et al., 2019). C-CAP comprises P1, WH2 domain, P2 and CARP domain and catalyzes nucleotide exchange of G-actin (Moriyama and Yahara, 2002; Dodatko et al., 2004; Quintero-Monzon et al., 2009; Makkonen et al., 2013; Nomura and Ono, 2013; Jansen et al., 2014; Kotila et al., 2018). Structural analysis and modeling of a tripartite complex composed of a ADF-H, ADP-actin and HFD showed that ADF/Cofilin binding to ADP-actin at the filament’s pointed end changes the conformation of the actin subunits to favor simultaneous docking of two HFD to both the ultimate and the penultimate actin subunit (Tanaka et al., 2018; Kotila et al., 2019). HFD bound to the penultimate subunit destabilizes the interaction between both subunits, whereby the ADF/Cofilin-bound ultimate subunit dissociates from the pointed end while being bound to HFD (Kotila et al., 2019). As mentioned above, ADP-G-actin binding interfaces of ADF-H from ADF/Cofilin (or Twinfilin) and HFD from CAP do not overlap (Paavilainen et al., 2008). Therefore, ADF/Cofilin stays bound to HFD via ADP-G-actin, in line with previous biochemical data showing that N-CAP interacts efficiently only with ADF/Cofilin-ADP-G-actin complexes (Moriyama and Yahara, 2002; Quintero-Monzon et al., 2009). Furthermore, using dye labeled ADF/Cofilin-decorated F-actin, it was shown that both mouse and yeast N-CAP associate with pointed ends (Kotila et al., 2019; Shekhar et al., 2019), where the HFD binding interfaces of two terminal actin subunits might be exposed (Kotila et al., 2019). Importantly, treatment of either Gelsolin- or CAPZ-capped ADF/Cofilin-decorated F-actin with mouse N-CAP or yeast CAP enhanced pointed end depolymerization 30 or 100 fold, respectively (Kotila et al., 2019; Shekhar et al., 2019). Moreover, in comparison to the steady state (bare) F-actin depolymerization, presence of N-CAP or yeast CAP together with ADF/Cofilin increased the rate of pointed end depolymerization up to 100 or 330 fold, respectively (Kotila et al., 2019; Shekhar et al., 2019). Strikingly, although treatment of bare F-actin with mouse N-CAP increased pointed end depolymerization 2–3 folds (Kotila et al., 2019), the full length yeast CAP displayed 7-fold increase, twice as efficient as in ‘ADF/Cofilin only’ condition (Shekhar et al., 2019). Finally, full length mouse and yeast CAP accelerates pointed end F-actin depolymerization in conditions with high concentrations of Profilin and G-actin, thus mimicking physiological conditions (Kotila et al., 2019; Shekhar et al., 2019). Based on these observations, the depolymerization rate at ADF/Cofilin-bound pointed end by mouse N-CAP and yeast CAP was calculated as 13 and 44 subunits/second, respectively (Kotila et al., 2019; Shekhar et al., 2019). These values approach the estimated rates of actin turnover *in vivo* (Miyoshi and Watanabe, 2013), implying that synergistic interaction of ADF/Cofilin and CAP is the main driver of rapid F-actin depolymerization at pointed ends (Kotila et al., 2019; Shekhar et al., 2019). Twinfilin, another ADF-H-containing ABP involved in G-actin sequestering and barbed end capping, has been recently shown to enhance barbed end and pointed end F-actin depolymerization in presence of yeast CAP 3 and 17 fold, respectively (Goode et al., 1998; Paavilainen et al., 2007; Johnston et al., 2015). Despite the fact that mouse Twinfilin and CAP failed to jointly induce pointed end depolymerization, F-actin disassembly at barbed end was activated to the same extent (threefold) as with their yeast homologs (Hilton et al., 2018). Although, the pointed end disassembly mechanism might be similar to the one of ADF/Cofilin, how exactly CAP induces Twinfilin-mediated barbed end depolymerization needs further clarification.

In addition to CAP’s actin depolymerization activity inherent to N-CAP, C-CAP can promote dissociation of ADF/Cofilin from ADP-G-actin and catalyze nucleotide exchange on G-actin (Balcer et al., 2003; Mattila et al., 2004; Chaudhry et al., 2007; Quintero-Monzon et al., 2009; Nomura et al., 2012; Makkonen et al., 2013; Jansen et al., 2014). Mechanistically, this can be explained by the finding that ADP-G-actin binding interface of CARP does not overlap with that of HFD (Kotila et al., 2018, 2019). Thus, after removal of the ADF/Cofilin-bound terminal actin subunit from pointed ends by HFD, ADP-G-actin is transferred to the CARP domain, which has high affinity for ADP-G-actin (Moriyama and Yahara, 2002; Mattila et al., 2004; Kotila et al., 2018). Intriguingly, CARP binding to ADP-G-actin induces a sterical clash with ADF-H, and the WH2 binding of G-actin might further destabilize the association of ADF-H and ADP-G-actin (Balcer et al., 2003; Mattila et al., 2004; Chaudhry et al., 2010, 2014; Kotila et al., 2018). As a result, HFD as well as ADF/Cofilin dissociates from ADP-G-actin, which becomes subsequently recharged with ATP presumably by CARP in cooperation with WH2 (Chaudhry et al., 2014; Jansen et al., 2014; Kotila et al., 2018). Biochemical assays confirm the requirement of complete mouse and yeast C-CAP segment for nucleotide exchange of ADF/Cofilin-bound ADP-G-actin, implying that WH2 and CARP domains must be connected for efficient activity in G-actin recharging (Chaudhry et al., 2014; Jansen et al., 2014). In the next step, the only domain of CAP that has comparably higher affinity to ATP-G-actin, i.e., WH2 domain (Chaudhry et al., 2010), might release the polymerization competent G-actin to the surrounding or transfer it to ATP-G-actin-binding proteins such as Profilin (Bertling et al., 2007; Makkonen et al., 2013). Future studies will show which of those mechanisms occurs *in vivo*.

#### F-Actin Severing

F-actin severing by ADF/Cofilin enhances actin turnover, at least in part, by increasing the number of filaments’ pointed ends that can be depolymerized (Pollard, 2016). Based on real time assessment of immobilized F-actin sparsely labeled with biotin, it has been proposed that yeast and mouse CAP can enhance F-actin severing activity of ADF/Cofilin by direct interaction with ADF/Cofilin-decorated F-actin segments (Normoyle and Brieher, 2012; Chaudhry et al., 2013; Jansen et al., 2014). However, follow up studies using F-actin immobilized only via barbed end, thereby leaving the complete filament free in solution, showed that CAP effect on ADF/Cofilin-mediated F-actin severing is very modest when compared to F-actin depolymerization (Kotila et al., 2019; Shekhar et al., 2019). To which extent CAP accelerates ADF/Cofilin-mediated F-actin severing *in vivo* awaits further studies.

#### INF2 Inhibition

In addition to the aforementioned functions, CAP controls actin dynamics via its inhibitory function towards the F-actin assembly factor INF2 (Mu et al., 2019, 2020). Specifically, binding of lysine-acetylated actin (KAc-actin) containing CAP to INF2’s regulatory regions, i.e., DID (Diaphanous inhibitory domain) and DAD (Diaphanous autoregulatory domain), keeps INF2 in an inactive state (Mu et al., 2019, 2020). INF2 has been previously implicated in F-actin assembly at contact sites between the endoplasmic reticulum (ER) and mitochondria, which is relevant for mitochondrial recruitment of Dynamin-related protein 1 (DRP1), mitochondrial fission and calcium transfer from ER to mitochondria (Korobova et al., 2013; Chakrabarti et al., 2018). Interestingly, yeast CAP has been recently implicated in mitochondria morphology and function, too (Chen et al., 2019). This study revealed an interaction of yeast CAP with DRP1 and showed that CAP deletion caused elongated-hyperfused mitochondria associated with lower reserved respiration capacity. Hence, these data suggested a pro-fission activity for yeast CAP. CAP may control mitochondria morphology and function via regulating INF2 activity. However, apart from INF2, ADF/Cofilin has been implicated in mitochondrial DRP1-recruitment, mitochondrial dynamics and function, too (Chua et al., 2003; Klamt et al., 2009; Rehklau et al., 2012, 2017; Hoffmann et al., 2019), thereby offering an additional mode of CAP action on mitochondria.

#### G-Actin Sequestration

Based on earlier *in vitro* studies showing that CAP inhibits spontaneous actin polymerization, it was concluded that CAP acts as an actin sequestering protein (Gieselmann and Mann, 1992; Freeman et al., 1995; Mattila et al., 2004; Chaudhry et al., 2007; Peche et al., 2012; Nomura and Ono, 2013). Although CAP has high affinity to ADP-G-actin (*Kd* 0.02–0.05 μM; Mattila et al., 2004; Makkonen et al., 2013), it also possesses fast catalytic activity towards nucleotide exchange on G-actin (Chaudhry et al., 2010, 2013; Jansen et al., 2014). Taking into consideration that compared to Profilin (*Kd* 0.1 μM) or WH2 domain containing proteins CAP possesses relatively poor ATP-G-actin affinity (*Kd* 1.5 μM; Vinson et al., 1998; Chaudhry et al., 2010; Carlier and Shekhar, 2017), it is less likely that CAPs can efficiently sequester G-actin in a complex milieu of the cell. However, further investigations are needed to clarify CAP’s G-actin sequestering function and its implication *in vivo*.

#### Oligomerization as Necessity

Using different methods such as gel filtration or analytical ultracentrifugation, previous studies identified yeast, mouse and human CAP as part of a 600 kDa complex containing six CAP and six G-actin, thereby suggesting formation of CAP hexamers (Moriyama and Yahara, 2002; Balcer et al., 2003; Quintero-Monzon et al., 2009; Jansen et al., 2014; Mu et al., 2019). Furthermore, negative staining electron microscopy revealed that yeast and mouse N-CAP segments including OD and HFD form a wheel-like appearance with six symmetrical protrusions. Interestingly, within each of this protrusion one could dock only one HFD, thus forming again a hexameric structure. Additionally, other studies stated that human N-CAP form tetramers (Purde et al., 2019). Whether this discrepancy is due to different experimental conditions needs further clarification. The most N-terminal coiled-coil OD has been implicated in oligomerization, since yeast CAP lacking OD could only dimerize (Quintero-Monzon et al., 2009), most likely via CARP domain (see CARP domain section). Corroborating this result, human N-CAP lacking OD formed mostly monomers, albeit to a small extent also dimers (Purde et al., 2019). Importantly, deletion of OD in yeast CAP reduced ADF/Cofilin-mediated F-actin turnover *in vitro* and caused defects in cell growth, morphology and actin organization *in vivo* (Quintero-Monzon et al., 2009). Interestingly, a substitution of OD of human N-CAP with artificial ODs, capable of forming either dimers or trimers, potentiated cofilin mediated F-actin depolymerization and severing better with increasing level of oligomerization (Purde et al., 2019). Likewise, dimerizing HFD of human CAP with glutathione-S-transferase partially rescued a 20-fold reduction in pointed end depolymerization of ADF/Cofilin-decorated F-actin by OD lacking N-CAP (Kotila et al., 2019). In summary, these observations strongly suggest that higher-order oligomerization state is necessary for CAP’s function in actin dynamics.

#### Concluding Remarks on CAP Function in Actin Dynamics

Taken together, studies of the last decade drastically changed our view of CAP’s contribution to actin dynamics. Instead of being G-actin sequestering factor or passive player in actin turnover, these studies unraveled important functions for CAP in almost all steps of actin dynamics including F-actin depolymerization, F-actin severing, G-actin recharging and F-actin polymerization, making CAP an essential player in actin dynamics regulation (Chaudhry et al., 2013; Jansen et al., 2014; Johnston et al., 2015; Kotila et al., 2019; Mu et al., 2019; Shekhar et al., 2019).

## Cellular, Developmental and Physiological Functions

### Cellular Functions in Lower Eukaryotic Organisms

Cyclase-associated protein functions in budding yeast have been outlined above. Briefly, studies in *S. cerevisiae* revealed a role for CAP in RAS2-cAMP signaling (Fedor-Chaiken et al., 1990; Field et al., 1990), which may depend on CAP’s stimulatory effect on post-translational RAS2 modification (Lila and Drubin, 1997). Furthermore, an actin regulatory activity of CAP was first described in yeast (Vojtek et al., 1991; Balcer et al., 2003; Mattila et al., 2004; Bertling et al., 2007). Apart from *S. cerevisiae*, cellular CAP functions have been studied in the soil-dwelling amoeba *Dictyostelium* (*D*.) *discoideum*. CAP inactivation in *D. discoideum* caused defects in cell polarization, F-actin organization and phototaxis, thereby confirming the relevance of CAP for various cellular functions. In *D. discoideum*, CAP is relevant for both cAMP signaling and actin cytoskeleton regulation (Noegel et al., 2004; Sultana et al., 2012), similar to the yeast CAP. Furthermore, actin-regulating activity was located in CAP’s C-terminal region in *D. discoideum*, and it was inhibited by phosphatidylinositol 4,5-bisphosphate (PIP2) (Gottwald et al., 1996). In migrating amoeba CAP, was located at anterior and posterior plasma membrane regions and enriched in leading fronts upon chemotactic stimulation, thereby suggesting a function in PIP2-dependent actin cytoskeleton regulation. Moreover, these observations suggested important developmental functions for CAP in multicellular organisms.

### Developmental and Physiological Functions in Invertebrates

*Drosophila melanogaster* only possesses a single CAP homolog (Ono, 2013), which has been identified simultaneously in two independent screens of mutant fly strains and termed Capulet and Act up, respectively (Baum et al., 2000; Benlali et al., 2000). In this review we stick to the term Capulet, which has been used more frequently in literature. Both screens unraveled Capulet as an important F-actin regulator that controls developmental patterning processes via actin-dependent mechanisms (Stevenson and Theurkauf, 2000). The first study found Capulet in a screen for oocyte polarity defects (Baum et al., 2000). During oogenesis, Capulet controls spatial F-actin assembly that is relevant for microtubule organization and, hence, for the asymmetric distribution of cell polarity determinants. The second study found antagonistic F-actin functions in the eye disk for Capulet and Chickadee, the fly homolog of Profilin. Although F-actin levels were increased in Capulet and reduced in Chickadee mutants (Benlali et al., 2000), both strains displayed similar defects in cell shape changes during eye development. These changes are relevant for the establishment of the eye disk morphogenetic furrow that restricts Sonic hedgehog (SHH) signaling and prevents premature photoreceptor differentiation. Consequently, SHH signaling and photoreceptor differentiation was less confined in mutant eyes (Benlali et al., 2000), demonstrating that actin-dependent cell morphological changes controlled by Capulet and Chickadee govern intercellular signaling cascades during development.

Since these pioneering, first genetic studies in multicellular organisms, several other important developmental functions have been unraveled for Capulet. It counterbalances F-actin assembly promoted by the Ena/VASP (vasodilator-stimulated phosphoprotein) protein Enabled at apical adherens junctions of follicular epithelia cells that cover oocytes during oogenesis (Baum and Perrimon, 2001). In this process, Capulet cooperated with the tyrosine kinase ABL, which interacts via its SH3 domain with Capulet’s proline-rich motif (Freeman et al., 1996). A function for Capulet in F-actin regulation at apical adherens junctions has been described also for wing epithelial cells (Major and Irvine, 2005). In these cells, Capulet acts downstream of Notch signaling in establishing a boundary between different cell populations and, hence, in wing compartmentalization. Boundary formation and wing compartmentalization was preserved in ABL mutants, demonstrating that ABL is not relevant for adherens junctions in wing epithelial cells. Hence, Capulet does not necessarily require ABL to be functional in epithelia cells. However, similar to follicular epithelia, a cooperation of Capulet and ABL has been reported in neuronal growth cones, which are dynamic and F-actin-enriched structures that navigate axons through the developing central nervous system (Wills et al., 2002). Specifically, Capulet and ABL interact in growth cone repulsion downstream of the secreted guidance cue Slit and its receptors of the Roundabout family, a pathway that spatially controls midline crossing of axons. Interestingly, Chickadee has been identified as another interaction partner of ABL in growth cones (Wills et al., 1999). Different from Capulet mutants, Chickadee mutants displayed growth cone arrest phenotype, suggesting opposing functions for both ABP in growth cones, similar to the eye disk (Benlali et al., 2000). While this study linked Capulet to F-actin regulation during neuron differentiation, another study described abnormal F-actin aggregates upon Capulet inactivation in differentiated neurons (Medina et al., 2008). In line with a biochemical function in releasing ADF/Cofilin from actin complexes (Johnston et al., 2015; Kotila et al., 2019; Shekhar et al., 2019), neurons from Capulet mutants displayed rod-like structures consisting of actin and ADF/Cofilin. Interestingly, similar ADF/Cofilin-actin rods have been found together with amyloid deposits and neurofibrillary tangles in brains from Alzheimer’s Disease (AD) patients as well as in abnormal actin aggregates termed Hirano bodies, which have been reported for AD and Parkinson’s disease (Minamide et al., 2000; Heredia et al., 2006; Gallo, 2007). Hence, defects in CAP-dependent neuronal actin dynamics may contribute to the pathology of human neurodegenerative diseases (see below).

Different from *D. melanogaster* and most other invertebrates, the nematode *C. elegans* expresses two CAP isoforms that have been termed CAS-1 and CAS-2, because the abbreviation CAP has been used already for actin capping proteins (Ono, 2013). CAS-1 and CAS-2 are encoded by distinct genes and differ in their expression pattern (Nomura et al., 2012; Nomura and Ono, 2013). While CAS-1 is abundant in muscle tissue, CAS-2 expression is restricted to non-muscle cells. In line with a conserved function in actin cytoskeleton regulation, CAS-1 binds G-actin and enhances exchange of actin-bound nucleotides (Nomura et al., 2012). Further, it promotes F-actin turnover in the presence of UNC-60B, the muscle-specific ADF/Cofilin homolog in *C. elegans*, which has been identified as an essential regulator of sarcomere F-actin organization in the larval body wall muscle (Ono et al., 1999, 2003). Genetic CAS-1 inactivation caused developmental arrest at larval stages, immobility as well as a severe F-actin disorganization in larval body wall muscles, while F-actin structures appeared normal in non-muscle tissues (Nomura et al., 2012). This study revealed a specific function for CAS-1 in striated muscles, and it strongly suggested a cooperative activity of CAS-1 and UNC-60B in sarcomere F-actin organization during myofibril differentiation, similar to a model that has been proposed for the mammalian homologs Cofilin2 and CAP2 (Kepser et al., 2019).

The second CAP homolog CAS-2 has been identified in *C. elegans* just a few years ago (Nomura and Ono, 2013). *In vitro* studies unraveled a primary function for CAS-2 in nucleotide exchange on G-actin and, hence, in F-actin assembly, which depends on β-sheets located in its CARP domain and on a C-terminal dimerization motif (Nomura and Ono, 2013; Iwase and Ono, 2016, 2017). CAS-2 has been shown to antagonize F-actin depolymerization and G-actin sequestration by the ADF/Cofilin homolog UNC-60A (Nomura and Ono, 2013). UNC-60A is widely expressed in non-muscle tissues and essential for embryonic cytokinesis and assembly of contractile actin networks in the somatic gonad (Ono et al., 2003, 2008), and CAS-2 may have similar important *in vivo* functions. However, the precise CAS-2 expression pattern has not been resolved to date and, due to the lack of CAS-2 mutant worms, its developmental and physiological functions remained unknown.

### Developmental and Physiological Functions in Vertebrates

Unlike most invertebrates, vertebrates express two CAP isoforms with different expression patterns (Ono, 2013). In most vertebrate species investigated to date, one of these isoforms (CAP1) is broadly expressed, while expression of the second (CAP2) is restricted to a limited number of tissues, including heart, skeletal muscle and brain as well as - in lower amounts - in skin, testes and lung (Bertling et al., 2004; Peche et al., 2007). This led to the suggestion that vertebrate CAPs evolved cell type-specific functions (Ono, 2013), similar to the muscle-specific function of CAS-1 in *C. elegans* (Nomura et al., 2012). Although abundance in striated muscles has been reported for CAP2 in several species from frog (*Xenopus laevis*) and zebrafish (*Danio rerio*) to mammals (Bertling et al., 2004; Peche et al., 2007; Wolanski et al., 2009; Effendi et al., 2012), its function in striated muscles has been studied only in mice, in which systemic inactivation caused a dilated cardiomyopathy (DCM) together with impaired cardiac conduction (Peche et al., 2012; Stockigt et al., 2016). These defects might be caused by disturbed sarcomere organization and/or by reduced cooperativity of calcium-induced force generation, which have been both shown for isolated CAP2 mutant myofibrils (Peche et al., 2012). Impaired heart physiology in CAP2 mutant mice has been confirmed in an independent study that also included a mutant strain with specific CAP2 inactivation in cardiac muscle cells (Field et al., 2015). This study further reported that cardiac conduction defects can culminate in a complete heart block, which likely caused increased lethality of systemic and cardiomyocyte-specific mutants (Peche et al., 2012; Field et al., 2015). Of note, DCM and cardiac conduction defects have been recently associated with CAP2 mutations in humans (Aspit et al., 2019).

Gene expression analyses revealed an upregulation of fetal genes in hearts from CAP2 mutant mice prior to the manifestation of clinical symptoms (Xiong et al., 2019). Interestingly, target genes of the transcription factor serum response factor (SRF) including genes encoding for α-actin isoforms were overrepresented among upregulated genes. Pharmacological inhibition of SRF activity not only normalized expression of SRF downstream targets, but also prolonged normal cardiac function and survival in CAP2 mutant mice, thereby demonstrating that SRF dysregulation contributed to heart defects in mutant mice. Myocardin-related transcription factor (MRTF) is an important co-activator of SRF, which is sequestered by G-actin and promotes expression of cytoskeleton-related genes upon release from G-actin complexes (Olson and Nordheim, 2010; Esnault et al., 2014). CAP2 may control SRF-dependent gene expression in cardiomyocytes via regulating availability of G-actin, in line with the elevated nuclear MRTF levels in CAP2 mutant hearts. These findings support an interesting model, in which actin regulators such as CAP2 are developmentally and physiologically relevant not only by controlling local F-actin dynamics in subcellular structures, but also by governing gene expression.

While these studies unequivocally demonstrated the relevance of CAP2 for heart physiology in mice and human, CAP2 seems to be dispensable for heart development in embryogenesis (Peche et al., 2012; Field et al., 2015). Conversely, a recent study identified an important function for CAP2 in myofibril differentiation during skeletal muscle development (Kepser et al., 2019). Specifically, CAP2 inactivation delayed the sequential exchange of α-actin isoforms from smooth muscle and cardiac α-actin to skeletal muscle α-actin during early postnatal development. This delay coincided with the onset of motor function deficits and histopathological changes characterized by a high frequency of displaced myofibrils termed ring fibers (Kepser et al., 2019). A very similar delay in the ‘α-actin switch’ has been reported for mutant mice lacking Cofilin2 (Gurniak et al., 2014), suggesting that CAP2 and Cofilin2 cooperate in myofibril actin cytoskeleton differentiation, similar to CAS-1 and UNC-60B in *C. elegans* (Nomura et al., 2012). Notably, Cofilin2 mutant mice displayed skeletal muscle phenotypes similar to myopathies described for human patients with *CFL2* mutations (Agrawal et al., 2007; Ockeloen et al., 2012; Ong et al., 2014). It is therefore tempting to speculate that mutations in the human CAP2 gene may cause skeletal muscle defects, too.

Deletions of the short arm of chromosome 6 that include the human CAP2 gene, have been described in a rare developmental disorder named 6p22 syndrome (Davies et al., 1999; Bremer et al., 2009; Celestino-Soper et al., 2012; Di Benedetto et al., 2013). This syndrome is characterized by developmental delays, heart defects as well as autism spectrum disorders (ASD) symptoms, which have been associated with synaptic defects (Bourgeron, 2015). Because CAP2 mutant mice displayed a delay in motor functions during postnatal development together with heart defects (Peche et al., 2012; Field et al., 2015; Kepser et al., 2019), a contribution of CAP2 loss to 6p22 syndrome has been suggested. Notably, apart from striated muscles, CAP2 is abundant in brain and present in different brain areas including cerebral cortex and hippocampus (Bertling et al., 2004; Peche et al., 2007; Kumar et al., 2016; Pelucchi et al., 2020a). In differentiated neurons, CAP2 is present in postsynaptic compartments (dendritic spines) of excitatory synapses and located in the F-actin enriched region underneath the postsynaptic density (Pelucchi et al., 2020a). CAP2 inactivation differently affected neuron structure and dendritic spine morphology in cerebral cortex and hippocampus. While primary cortical neurons from CAP2 mutant mice showed an increase in dendrite complexity and spine density (Kumar et al., 2016), CAP2 downregulation in primary hippocampal neurons reduced dendritic arborization and enlarged spines, which was associated with decreased synaptic excitatory transmission and impaired synaptic plasticity (Pelucchi et al., 2020a). Interestingly, CAP2 function in spine morphology and synaptic plasticity required its ability to form disulfide cross-linked homodimers, which were mediated by cysteine-residues at position 32 (C32). C32-dependent covalent dimerization of CAP2 was crucial for interaction with Cofilin1 (Pelucchi et al., 2020a), a key actin regulator in dendritic spines that controls spine morphology as well as different forms of synaptic plasticity, including long-term potentiation (LTP) and long-term depression (LTD; Rust, 2015). The mutation of the C32 of CAP2 significantly decreases, but not completely eliminates, CAP2 self-association and the binding to Cofilin1, suggesting that CAP2 can still form oligomers that are able to interact with Cofilin1. Yet, the lack of C32 covalent CAP2 dimers leads to a loss of function of CAP2/Cofilin1 complex on actin depolymerization, emphasizing the importance of C32 disulfide bond formation for the CAP2 function (Pelucchi et al., 2020a). In Cofilin1 mutant mice, mature spines were strongly enlarged in hippocampal and striatal neurons and LTD, which is associated with spine shrinkage or retraction, was not inducible (Rust et al., 2010; Wolf et al., 2015; Zimmermann et al., 2015), suggesting that Cofilin1 is relevant for F-actin disassembly in mature spines. Instead, during the early phase of LTP, Cofilin1 is recruited into dendritic spines where it may promote F-actin assembly that is required for spine expansion (Bosch et al., 2014). CAP2 may have a crucial role in the LTP-induced enrichment of Cofilin1 in spines: it has been shown that the C32-dependent CAP2 covalent dimerization and association to Cofilin1 are triggered by LTP and are required for LTP-induced Cofilin1 translocation into spines, spine remodeling and the potentiation of synaptic transmission (Pelucchi et al., 2020a). Redox-regulated disulfide bond formation represents an important post-translational control mechanism employed by several proteins, such as transcription factors, signaling proteins and cytoskeletal components to adjust their functional activity when reactive oxygen species (ROS) start to accumulate (Cremers and Jakob, 2013). Indeed, it is becoming clear that ROS are not simply toxic species, but are often transiently and locally produced as part of signaling pathways (Schippers et al., 2012; Cremers and Jakob, 2013). In neuronal cells, ROS generation has been also implicated in plasticity events (Bórquez et al., 2016), since it has been shown that the production of superoxide anion radical is required for the full expression of LTP and for memory tasks (Massaad and Klann, 2011). In light of these considerations, it might be possible that LTP-triggered changes in the redox balance could trigger the disulfide bond formation of CAP2 covalent dimers.

Together, these data implicated CAP2 in cellular processes that are believed to be fundamental for learning and memory (Holtmaat and Svoboda, 2009), and they suggested that CAP2 loss may contribute to ASD symptoms in 6p22 syndrome. Interestingly, a C29-dependent mechanism in covalent dimer formation relevant for F-actin and Cofilin1 binding has been also reported for CAP1 in rat mesangial cells (Liu et al., 2018). However, the physiological relevance of CAP1 covalent dimerization and Cofilin1 interaction has not been studied in neurons to date. Unlike C32 in CAP2, C29 in CAP1 is not conserved in humans, thereby limiting the relevance of CAP1 covalent dimerization for synaptic function.

Immunoblot analysis and *in situ* hybridization revealed broad CAP1 expression in mice, both during development and in adulthood (Bertling et al., 2004; Peche et al., 2007). However, only very little is known about its developmental and physiological functions, also because appropriate mouse models were missing. Transcription activator-like effector nuclease (TALEN)-engineered systemic CAP1 mutant mice have been reported just recently, but these mutants died at embryonic day 16.5, and their developmental defects have not been analyzed yet (Jang et al., 2019). Instead, heterozygous mutants with substantially reduced CAP1 protein levels were viable and showed defects in lipoprotein metabolism. Specifically, CAP1 was identified as an interaction partner of proprotein convertase subtilisin/kexin type-9 (PCSK9), which induces internalization and lysosomal degradation of low-density lipoprotein (LDL) receptor (LDLR). PCSK9 thereby enhances serum levels of LDL and LDL cholesterol, and it emerged as a valuable therapeutic target for atherosclerotic cardiovascular diseases (Burke et al., 2017). Mechanistically, PCSK9-binding of CAP1 promotes Caveolin-1-dependent endocytosis and lysosomal degradation of PCSK9-LDLR complexes (Jang et al., 2019). This pathway was impaired upon CAP1 inactivation, and heterozygous mice displayed increased LDLR levels and consequently reduced serum levels of LDL and LDL cholesterol. Hence, modulation of CAP1 activity may provide a novel therapeutic avenue for atherosclerosis and other cardiovascular diseases (Dron and Hegele, 2020). Apart from its function in lipoprotein metabolism, no other *in vivo* function has been reported for CAP1 to date, underlying the exigency of a conditional mouse model, which would also allow to test whether or not CAP1 is relevant for cytokine signaling, inflammation, adipose biology, coronary artery disease, chronic obstructive pulmonary disease or renal disease as suggested by recent studies (Lee et al., 2014; Xie et al., 2014; Munjas et al., 2017; Munjas et al., 2020).

### Potential Implication in Human Diseases

As a major cytoskeletal component in eukaryotic cells, F-actin is involved in a variety of cellular processes. Together with actin motor proteins, it constitutes the primary machinery for the generation of protrusive and contractile forces (Castellano et al., 2001; Amberg et al., 2012). F-actin dynamic is relevant for cell migration, phagocytosis and membrane trafficking. In addition, actin is the target of executioner Caspases during apoptosis, and experiences oxidative damage when cellular stress occurs (Davidson and Wood, 2016). In this framework, ABP are critical for the precise control of the actin cytoskeleton, since they are responsible for forming the F-actin structures at the right place and time within the cell. In light of these considerations, the actin cytoskeleton and, thereby, ABP play key roles in many aspects of human health, ranging from embryonic development to aging, and are implicated in several diseases and pathological processes including cancer metastasis, wound repair, inflammation or neurodegenerative disorders (Davidson and Wood, 2016; Lai and Wong, 2020; Pelucchi et al., 2020b). As far as concern CAP1 and CAP2, potential roles have been described for various human pathologies apart from the already mentioned contributions of CAP1 to atherosclerosis or other cardiovascular diseases and of CAP2 to heart diseases and 6p22 syndrome.

#### CAP1 at the Crossroads of Metabolism and Cancer

Several publications reported altered CAP1 expression in a growing list of human cancers that include glioma, oral squamous cell carcinoma as well as breast, pancreatic, liver, lung and epithelial ovarian cancer. CAP1 is a protein relevant for cell migration and, thereby, presumably in metastasis formation. Indeed, dynamic actin cytoskeletal rearrangement, based on repeated cycles of F-actin turnover, is the primary driving force of cell migration and cancer cell invasiveness (Hall, 2009; Fife et al., 2014). Overexpression of CAP1 may have significant clinical implications as a diagnostic/prognostic factor for lung cancer (Tan et al., 2013), esophageal squamous cell carcinoma (Li et al., 2013), epithelial ovarian cancer (Hua et al., 2015) and glioma (Bao et al., 2016; Fan et al., 2016). The loss of CAP1 expression affects the breast cancer cell cycle (Yu et al., 2014), retards the glioma cells proliferation (Bao et al., 2016; Fan et al., 2016) and inhibits cell cycle progression in epithelial ovarian cancer cells (Hua et al., 2015).

CAP1 overexpression in hepatocellular carcinoma specimens correlates with tumor metastasis. Moreover, CAP1 co-localizes with actin in the leading edge of lamellipodia in hepatocellular carcinoma cells (Liu et al., 2014). CAP1 down-regulation impairs cells migration in hepatocellular carcinoma cells (Liu et al., 2014), in esophageal squamous cell carcinoma (Li et al., 2013), in breast cancer cells (Yu et al., 2014) and in glioma cells (Bao et al., 2016; Fan et al., 2016). However, the role for CAP1 in human cancers and in cell migration is still controversial, with mounting evidence suggesting a role that is dependent on the type or even subtype of cancer. CAP1 knockdown impaired F-actin dynamics, which in most cells leads to reduced cell motility. However, depletion of CAP1 in HeLa cells, while causing reduction in dynamics, actually caused increased cell motility through activation of cell adhesion signals (Zhou et al., 2014a). In metastatic breast cancer cells, depletion of CAP1 stimulated both the invasiveness and cell proliferation, while in non-metastatic MCF-7 cancer cells it actually had opposite effects (Zhang and Zhou, 2016).

Pancreatic cancer has the worst prognosis among cancers due to the difficulty of early diagnosis and its aggressive behavior. CAP1 was found upregulated in pancreatic cancer xenografts transplanted into immuno-deficient mice, and CAP1-positive tumor cells in clinical specimens correlated with the presence of lymph node metastasis and with the poor prognosis of patients (Yamazaki et al., 2009). CAP1 inactivation resulted in reduced lamellipodium formation, cell motility and invasion (Yamazaki et al., 2009). In another study, no changes in CAP1 expression in pancreatic cancer lines have been reported, but an increase in CAP1 phosphorylation at serine residues S308/S310 has been detected (Wu et al., 2019). CAP1 phosphorylation at this tandem phospho-site controls binding of Cofilin1 and actin and provide a mechanism that controls F-actin dynamics (Zhou et al., 2014b). The phosphorylation mutants showed defects in alleviating the elevated focal adhesion kinase (FAK) activity and enhanced focal adhesions in the CAP1 knockdown cells (Zhang et al., 2020). Overall, these results support the idea that transient CAP1 phosphorylation controls F-actin dynamics and cell adhesion. Interestingly, glycogen synthase kinase 3 (GSK3), which was reported to be hyper-activated in pancreatic cancer, can phosphorylate CAP1 (Zhou et al., 2014b). Disrupting CAP1 phospho-regulation via GSK3 inhibition or expressing phospho-site mutants compromised CAP1 functions in alleviating enhanced stress fibers and in rescuing invasiveness of CAP1-knockdown pancreatic cancer cells. These data suggest that transient CAP1 phosphorylation is relevant for the control of pancreatic cancer cell invasiveness (Wu et al., 2019). The involvement of this tandem phospho-site has been reported also in breast cancer cells, in which CAP1 has a role in the invasiveness and in regulating proliferative transformation of cancer cells, with ERK (extracellular signal-regulated kinase) signaling playing pivotal roles in mediating both cell functions (Zhang and Zhou, 2016).

In addition to the above-described mechanism related to cell migration and metastasis formation, CAP1 can contribute to cancer pathogenesis as Resistin receptor. Human Resistin is primarily expressed in and secreted from monocytes (Patel et al., 2003). Resistin-mediated chronic inflammation can lead to obesity, atherosclerosis, and other cardiometabolic diseases. In addition to the toll-like receptor 4 (TLR4; Tarkowski et al., 2010), CAP1 has been identified as receptor for human Resistin. Resistin binding of CAP1 via its SH3 domain upregulates cAMP concentration, protein kinase A (PKA) activity, and NF-κB-related transcription of inflammatory cytokines. Even though several biochemical binding assays have demonstrated the direct interaction between CAP1 and Resistin (Lee et al., 2014), the biological mechanism underlying CAP1/Resistin association at the plasma membrane requires further investigations considering that CAP1 lacks a transmembrane domain. Such concern should be taken into account also in relation to the binding of CAP1 to caveolin-1 and PCSK9, which is implicated in the caveolae-dependent endocytosis and lysosomal degradation of the LDLR. Further studies addressing how CAP1 is associated to the membrane are necessary to fully understand CAP1 cellular function.

CAP1 mediates the inflammatory response triggered by Resistin both in cultured human monocytes and in white adipose tissue in humanized Resistin mice *in vivo* (Lee et al., 2014). In addition, Resistin increases chemokine production by fibroblast-like synoviocytes via CAP1 in synovial tissue, thus contributing to the pathogenesis of rheumatoid arthritis (Sato et al., 2017).

Considering Resistin’s ability to stimulate lipid uptake and atherosclerotic plaque progression, CAP1 and Resistin levels have been assessed in patients affected by coronary artery disease. The results revealed a significant increase in plasma Resistin levels and in CAP1 expression in peripheral blood mononuclear cells of coronary artery disease patients, suggesting that Resistin is able to exert its effects stronger on cells with up-regulated CAP1 (Munjas et al., 2017).

An increase in Resistin plasma levels and CAP1 expression in peripheral blood mononuclear cells has been reported in colorectal cancer patients (Mihajlovic et al., 2019). Resistin can also contribute to pancreatic cancer pathogenesis, since its levels are increased in pancreatic cancer patients and correlate positively with tumor grades. Moreover, the Resistin receptors CAP1 and TLR4 mediate the effects of Resistin on cancer cells through activation of STAT3 (signal transducer and activator of transcription 3) and are implicated in the resistance to chemotherapy (Zhang et al., 2019).

Obesity represents a major risk for developing several types of cancer, including breast cancer (Calle and Kaaks, 2004). Resistin is among the top modulated adipokines secreted by adipocytes under obesity-associated metabolic conditions and therefore represents a plausible soluble mediator in the link between obesity, metabolic complications and breast cancer via the binding to CAP1 (Rosendahl et al., 2018). In a study, CAP1 gene and Resistin gene variants were associated with increased risk of breast cancer among Mexican women (Munoz-Palomeque et al., 2018). CAP1 is expressed across a large panel of breast cancer cell lines and primary human tumor and high CAP1 expression is associated with poor tumor characteristics and impaired prognosis among breast cancer patients (Rosendahl et al., 2018). Low CAP1 tumor expression was associated with higher body fatness and worse survival outcomes in breast cancer patients (Bergqvist et al., 2020). Moreover, Resistin increases breast cancer metastasis potential through induction epithelial to mesenchymal transition, a process in which cancer cells lose their epithelial characteristics and gain mesenchymal-like features, and that these effects may be associated with CAP1 (Avtanski et al., 2019).

#### CAP2 Role in AD, Wound Repair and Cancer

We have mentioned above the importance of CAP2 for synaptic plasticity that is relevant for learning and memory. F-actin alterations in dendritic spines have been described in AD, the most common form of dementia characterized by synaptic dysfunction in the early stages of the pathogenesis (Pelucchi et al., 2020b). CAP2 levels and synaptic localization are specifically reduced in the hippocampus, but not in the cortex of AD patients. Interestingly, CAP2 levels are increased in the cerebrospinal fluid of AD patients, but not in subjects affected by frontotemporal dementia, indicating the specificity of the alteration for this form of dementia. Furthermore, in AD hippocampal synapses CAP2 covalent dimer levels are decreased and Cofilin1 association to CAP2 covalent dimer/monomer is altered. These data suggested the presence of an ineffective CAP2-Cofilin1 complex in AD hippocampal synapses, which may contribute to impaired structural plasticity in AD (Pelucchi et al., 2020a).

CAP2 could be also involved in wound repair since CAP2 mutant mice showed an altered wound healing response (Kosmas et al., 2015). CAP2 in murine and human skin is present in the nucleus, in the cytosol and in the cell periphery. The keratinocytes from CAP2 mutant mice showed reduced velocity and a delay in scratch closure. Moreover, in human wounds, CAP2 is also expressed in hyper-proliferative epidermis. The fibroblasts of CAP2 mutant mice develop extended protrusions, increased focal adhesions and showed slower migration velocity, thereby suggesting a model in which a stabilization of focal adhesions as well as a disruption of cell polarity impaired motility of CAP2-deficient cells (Kosmas et al., 2015). The formation of a dense meshwork of peripheral F-actin and the disruption of the cell polarity may also contribute to reduced cell motility necessary to promote the wound healing process (Kosmas et al., 2015).

Similar to CAP1, CAP2 has been implicated in cancer pathogenesis and particularly in invasiveness. CAP2 is overexpressed in different cancer and is an unfavorable biomarker for prognostic prediction for patients affected by breast cancer (Xu et al., 2016), epithelial ovarian cancer (Adachi et al., 2020), malignante melanoma (Masugi et al., 2015), gastric cancer (Li et al., 2020), and glioma (Saker et al., 2020). Furthermore, oligonucleotides array technology revealed CAP2 as one of the genes upregulated in early hepatocellular carcinoma (Chuma et al., 2003). CAP2 overexpression is observed in a stepwise manner during the hepatocellular carcinoma progression and, in the early stages, the invading tumor cells were CAP2-positive (Shibata et al., 2006). Indeed, CAP2 overexpression is considered a poor prognostic biomarker for hepatocellular carcinoma patients (Fu et al., 2015). Interestingly, CAP2 and actin co-localized in the leading edge of lamellipodia from hepatocellular carcinoma cells. CAP2 knockdown inhibited lamellipodia extension upon serum stimulation and decreased cell motility. These data suggest a role for CAP2 in F-actin dynamics at the leading edge of lamellipodia, which is a characteristic feature of motile cells. Moreover, the overexpression of CAP2 correlates with portal vein invasion and intrahepatic metastasis, indicating CAP2 involvement in promoting the invasive behavior of hepatocellular carcinoma cells (Effendi et al., 2012).

## Concluding Remarks

Actin dynamics is coordinated by multiple ABP and involved in a variety of cellular functions including muscle contraction, cell movement, intracellular transport, and transcriptional regulation within the nucleus. In this complex picture, CAP, an ABP conserved among eukaryotes, has acquired crucial roles. First described as a G-actin sequestering factor, more recently CAP has emerged as a molecular hub able to orchestrate F-actin depolymerization, G-actin recharging and F-actin severing. The existence of two CAP isoforms with relatively low homology (Yu et al., 1994) and different expression patterns in vertebrates, further increases the complexity. CAP1 is essential in most cell types, while CAP2 appears to have unique roles. Considering that most studies so far have been focused on CAP1, it will be important to investigate the function of CAP2 in specific cell types expressing high CAP2 levels.

In addition to the actin regulating activity, CAP1 has been identified as Resistin receptor and protein partner of PCSK9, thus highlighting the involvement of CAP1 in metabolic processes. Such novel biological function suggests that CAP could be not just an ABP, but it can be involved in multiple biological pathways. This hypothesis is supported by the involvement of CAP1 and CAP2 in the pathogenesis of different diseases.

How are CAP-mediated processes governed in cells? To address this issue, it will be crucial to integrate structural information and biological studies to identify post-translational modifications and signaling pathways controlling CAP activity. Such information will be relevant for a comprehensive characterization of CAP’s cellular functions and for understanding potential CAP dysregulation that may contribute to the pathogenesis of human diseases.

## Author Contributions

MR, SK, SP, and EM wrote the article. All authors contributed to the article and approved the submitted version.

## Conflict of Interest

The authors declare that the research was conducted in the absence of any commercial or financial relationships that could be construed as a potential conflict of interest.

## Abbreviations

AA: amino acid
ABD: actin-binding domain
ABL: abelson murine leukemia viral oncogene homolog 1
ABP: actin-binding protein
ABP1: actin-binding protein 1
AC: adenylyl cyclase
AD: Alzheimer’s disease
ADF: actin depolymerizing factor
ADF-H: actin depolymerizing factor homology domain
ASD: autism spectrum disorder
CAP: cyclase-associated protein
CAPZ: capping protein in Z band
CARP: CAP and RP2 domain
CAS-1/-2: CAP homologs in *C. elegans*
C-CAP: C-terminal part of CAP
DAD: diaphanous autoregulatory domain
DCM: dilated cardiomyopathy
DID: diaphanous inhibitory domain
DRP1: dynamin-related protein 1
ER: endoplasmic reticulum
ERK: extracellular signal-regulated kinase
F-actin: actin filament
FAK: focal adhesion kinase
G-actin: globular actin monomer
GSK3: glycogen synthase kinase 3
HFD: helical folded domain
INF2: inverted formin 2
KAc: lysine-acetylated
LDL: low-density lipoprotein
LDLR: LDL receptor
LTD: long-term depression
LTP: long-term potentiation
MRTF: myocardin-related transcription factor
N-CAP: N-terminal part of CAP
OD: oligomerization domain
P1: proline-rich motif 1
P2: proline-rich motif 2
PCSK9: proprotein convertase subtilisin/kexin type-9
PIP2: phosphatidylinositol 4,5-bisphosphate
PKA: protein kinase A
RAS2: ras-like protein 2
RP2: retinitis pigmentosa protein 2
SD: subdomain
SH3: Src homology 3 domain
SHH: sonic hedgehog
SRF: serum response factor
SRV2: suppressor of Ras2-Val19
STAT3: signal transducer and activator of transcription 3
TALEN: transcription activator-like effector nuclease
TLR4: toll-like receptor 4
UNC-60A/-60B: ADF/Cofilin homologs in *C. elegans*
VASP: vasodilator-stimulated phosphoprotein
WASP: Wiscott-Aldrich-Syndrome protein
WH2: WASP homology 2 domain.
